# Plasma Modification of Biomass-Based Starfish Catalysts for Efficient Biodiesel Synthesis

**DOI:** 10.3390/nano14151313

**Published:** 2024-08-04

**Authors:** Sungho Lee, Jeyoung Ha, Oi Lun Li

**Affiliations:** School of Materials Science and Engineering, Pusan National University, Busan 46241, Republic of Korea; behonder92@pusan.ac.kr (S.L.); vwpdudv@pusan.ac.kr (J.H.)

**Keywords:** biomass-based catalyst, starfish, biodiesel production, plasma modification, transesterification

## Abstract

This study investigated biodiesel production via the transesterification of grapeseed oil with plasma-modified biomass-based catalysts originating from starfish. Dried starfish was first converted into magnesium and calcium oxide through heat treatment and then further modified by plasma engineering to improve the catalyst’s surface area and active sites via zinc addition. The Zn content was added via plasma engineering in the ratios of starfish (Mg_0.1_Ca_0.9_CO_3_): ZnO varying from 5:1, 10:1, to 20:1. The structure and morphology of the catalyst were confirmed through XRD, SEM, and XPS analysis. After the Zn addition and activation process, the surface area and the basicity of the synthesized catalysts were increased. The plasma-modified catalyst showed the highest basicity at the ratio of 10:1. Based on HPLC analyses, the optimized biodiesel yield in transesterification demonstrated 97.7% in fatty acid conversion, and its catalytic performance maintained 93.2% even after three repeated runs.

## 1. Introduction

Global use of fossil fuels is increasing due to problems such as environmental pollution and fossil fuel depletion, and the need for sustainable energy is urgent [1]. Among various alternative energy sources, biodiesel stands out as a non-toxic, biodegradable, and renewable fuel [2]. Biodiesel production typically involves the transesterification of triglycerides from plant or animal oils to fatty acid esters and glycerol, a reversible reaction described in Equation (1). The process begins with activating the catalyst through heating to increase its surface area and reactivity. When triglycerides react with methanol, which is facilitated by the catalyst, it results in the formation of reactive methoxide anions. These anions react with triglyceride molecules, forming methyl esters and glycerol. This reaction requires an excess of methanol to shift the equilibrium towards biodiesel production [3].

Triglyceride + 3 Methanol ⇄ 3 Methyl Esters + Glycerol(1)

The wine industry generates huge quantities of grape marc, from which grape seed oil can be obtained. In Spain and India, 6900 and 120 kt of grapes are produced, respectively, demonstrating the significant potential of grape seed oil as a bioresource feedstock [4,5]. For edible grape seed oil, free fatty acids (FFAs) are removed because they adversely affect the oil’s flavor and stability due to their susceptibility to auto-oxidation [6].

On the other hand, biodiesel production costs are still relatively high, attributed to the cost of raw materials and complex processing routes [7,8]. A typical way to lower production costs is to incorporate catalysts, which accelerate the reaction rate and play an important role in biodiesel commercialization [1]. Catalysts can be broadly classified into two types: homogeneous catalysts and heterogeneous catalysts. Although homogeneous catalysts, including NaOH and KOH, provide a high production yield of biodiesel, these catalysts still suffer significant drawbacks, such as difficulty in separation between the catalysts and products due to the saponification reaction, as well as the post-treatment of the waste effluents [9]. On the other hand, the use of heterogeneous catalysts not only accelerates the reaction rate but also makes the separation process of the catalyst from the reaction product easy, which plays an important role in biodiesel commercialization [10,11].

Among many heterogenous catalysts, CaO has been studied with decent performance in transesterification [12,13,14,15]. Not only does CaO have a strong base strength and low solubility in methanol, but CaO-based materials are relatively low in price and can be supplied from various recycled/waste materials, such as eggshells [16] and crab shells [17]. Starfish, among other Ca-based wastes, have been identified as causing extreme losses to sea farmers and disturbing the marine ecosystem [18]. In addition, most starfish are supplied at a lower price or even treated as waste and incinerated [19]. When recycled as a waste catalyst, starfish can significantly reduce the capital cost of biodiesel production and solve environmental problems [18]. In addition, dried starfish consists of both magnesium and calcium [20], which has an advantage over other waste-based catalysts since it can be converted to CaO and MgO through annealing [21].

However, using a Ca-based catalyst alone in biodiesel production can lead to a leaching problem after transesterification, thus reducing the catalyst’s performance and durability [22]. There are several methods to solve these problems: (1) improve the stability of catalysts by using oxides such as SiO_2_ [23] and CeO_2_ [24], (2) increase yield by using alkali metals such as Li, Na, and K [25], and (3) improve both the stability and efficiency based on spinel structures with Zn- and Al-doped CaO, which was studied by [26]. In particular, zinc is used in many catalytic reactions because it is an inexpensive, stable, commercially efficient, and environmentally friendly catalyst [27,28]. Previous studies reported that Zn addition improves production yield by enhancing the alkalinity [29] and stability of CaO catalysts [30]. In general, Ca oxides are modified via ammonia or sodium hydroxide, adding complexity and cost to their production process [24,30,31,32]. This study, on the other hand, utilized an environmentally friendly and simple plasma process to modify biomass catalysts derived from starfish without the use of ammonia. The plasma-modified catalysts were prepared under various mole ratios of support material (starfish) to zinc at 5:1, 10:1, and 20:1, and comparisons were made regarding catalyst structure, chemistry, morphology, specific surface area, and surface alkalinity. Additionally, this study compared the yield of biodiesel production through the transesterification of grape seed oil, offering a method for producing eco-friendly biodiesel catalysts.

## 2. Experimental Section

### 2.1. Materials

Starfish (*Asterias rubens*) [20] from Beidaihe, China, were used as the biomass catalysts. Other chemicals, including Zn(CH_3_COO)_2_ 2H_2_O (zinc acetate dihydrate), methyl alcohol (99.9%), isopropyl alcohol (99.7%), and n-hexane (96%), were purchased from Duksan General Science. Grapeseed oil (Beksul Food Company, Seoul, Republic of Korea) was available in the local market. Methyl linoleate, methyl oleate, and methyl stearate for HPLC standard were purchased from Sigma Aldrich (St. Louis, MO, USA).

### 2.2. Preparation of Catalyst

Dried starfish was immersed in D.I. water for 48 h to remove impurities and then dried in an oven at 80 °C for 12 h. The washed starfish was ball-milled to prepare the catalyst powder (hereafter referred to as SF). SF was calcinated at 700 °C and 900 °C, respectively, for 2 h in an air atmosphere (hereafter referred to as SF700 and SF900) to convert magnesium calcite into calcium oxide and magnesium oxide.

Then, plasma modification was utilized to incorporate Zn into SF900 to form SFZnx catalysts. The Zn loading content was adjusted by adding 1, 2, and 4 mmol of zinc acetate, which corresponded to ratios of SF900: Zn of 20:1, 10:1, and 5:1 (referred to as SFZn1, SFZn2, and SFZn3). At first, the zinc acetate was stirred in 100 mL of D.I. water for 30 min. Then, both SF and zinc solutions were mixed in a 200 mL reactor to proceed with plasma engineering. This experiment used the plasma process for various catalytic syntheses using the same device as in our previous study [33,34,35]. Tungsten electrodes with ceramic tube insulation were placed at the reactor with a 1 mm space between electrodes and connected to a bipolar DC pulse power supply. Voltage, pulse frequency, and pulse width were fixed at 10 kV, 50 kHz, and 1.0 μs, respectively. Plasma discharge was performed for 10 min, followed by filtration and drying overnight. The synthesized powder was activated under annealing conditions at 780 °C for 2 h in an air atmosphere. In addition, a catalyst was prepared by hydrothermal synthesis [36,37] to evaluate the performance of the plasma process catalyst. The contents of this are shown in the Supplementary Materials.

### 2.3. Biodiesel Production via Transesterification

An amount of 1 g of grape seed oil and 1.75 mL of methyl alcohol were added to an 11 mL culture tube. An amount of 1 wt. % of catalyst (24 mg) was mixed with the prepared reactant (oil + methyl alcohol). The reactor was submerged in a water bath on a hot plate. This mixture was stirred vigorously at 1500 rpm using a magnetic bar at 68 °C for 12 h under atmospheric pressure.

To study reusability, catalyst recovery proceeded by collecting the catalysts with a centrifuge, washing the used catalyst with ethanol, and then heat treating at 300 °C for 2 h to eliminate the remaining ethanol, methanol, grapeseed oil, and biodiesel. Transesterification for catalyst recycling was conducted with the recovered catalysts. The conditions for transesterification were fixed as in the previous process. The recycle test was conducted for 2 more cycles.

### 2.4. Catalyst Characterization

The structure properties of catalysts were compared by X-ray diffraction (Rigaku Ultima IV X-ray diffractometer, Tokyo, Japan) with Cu Kα radiation operated at 40 kV and 40 mA. The surface chemical bonding states were characterized by an X-ray photoelectron spectrometer (XPS, JEOL, JPS-9010MC, Tokyo, Japan) at the Korea Basic Science Institute (KBSI, Busan, Republic of Korea) utilizing an X-ray gun emitting Al Kα monochromatic radiation (hν = 1486.6 eV). The quantitative analysis for the chemical composition of synthesized starfish-based catalysts was performed by an inductively coupled plasma optical emission spectrometer (PerkinElmer, Optima 8300, Waltham, MA, USA). The morphology of the prepared sample was investigated using a scanning electron microscope (SEM) at an acceleration voltage of 10 kV. Surface characteristics such as surface area, pore size, and pore volume of synthesized catalysts were investigated using ASAP2420 (Micromeritics, Norcross, GA, USA).

### 2.5. Catalytic Performance Test

HCl titration was processed to measure each catalyst’s basicity as follows: 300 mg of catalyst was placed with the addition of 10 mL of the 1% phenolphthalein solution in the beaker, and the mixture was stirred for 6 h to ensure proper mixing of the catalyst and the indicator solution. Then, the HCl solution was added slowly to the catalyst-indicator mixture while continuously stirring. The titration was continued until the indicator’s color change signaled the reaction’s endpoint. Lastly, the volume of HCl solution consumed was recorded and calculated to determine the basicity of the catalyst.

High-Performance Liquid Chromatography (HPLC, LC-20A, Kyoto, Japan) was conducted with a liquid chromatography device equipped with a Refractive Index Detector (RID-20A, Shimadzu, Kyoto, Japan) and a Pursuit 5 C18 column (250 × 4.6 mm with 5 μm particle size). The mobile phase was composed of 85% methanol, 10% isopropyl alcohol, and 5% n-hexane. The oven temperature and flow rate were 40 °C and 0.5 mL/min, respectively. The biodiesel yield was calculated by comparing the amount of methyl ester produced with the representative fatty acids contained per 1 g in grape seed oil (Table 1) to calculate each methyl ester [38]. Biodiesel yield was determined according to Equation (2) [39,40].

Yield of Biodiesel (%) = (Mass of methyl ester)/(Mass of grapeseed oil) × 100 (2)

## 3. Results and Discussion

### 3.1. Morphology and Textural Properties

**Figure 1:**
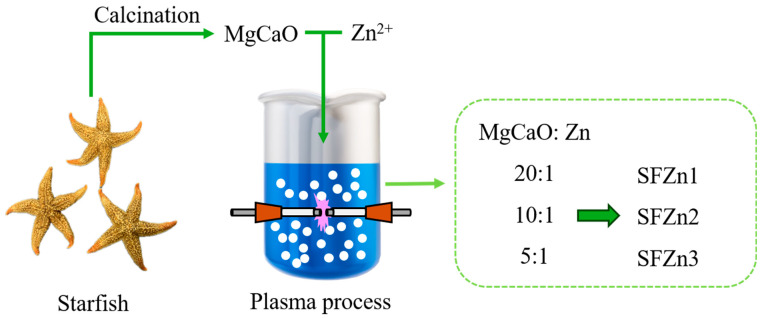
The schematic of plasma modification via the plasma process.

The schematic of the plasma modification of the Zn-added SF catalysts is shown in Figure 1. Starfish is composed of MgCO_3_ and CaCO_3_ (Mg_0.1_Ca_0.9_CO_3_), which was converted to MgO and CaO through heat treatment. Subsequently, SFZnx, a catalyst in which Ca, Mg, and Zn are mixed, was synthesized through a plasma process. The reaction equations for the total process are as follows [28,41]:

MgCO_3_ + CaCO_3_ → MgO + CaO + 2CO_2_ (3)

CaO + MgO + 2H_2_O → Ca(OH)_2_ + Mg(OH)_2_ (4)

Magnesium reacts insignificantly with water, and the initial magnesium concentration of the dried starfish was not high. As shown in Figure S1, most of the MgO remained unresponsive.

With plasma discharge in the water, H_2_O_2_ is produced through the following reactions where · refers to the radical, * refers to the excited atom, and e^−^ is the electron [42].

H_2_O → H_2_O·^+^ + e·^−^ (5)

H_2_O·^+^+ H_2_O → H_3_O·^+^ + ·OH (6)

H_2_O → H_2_O* → ·OH + ·H (7)

e·^−^ + OH^−^ → ·OH + e^−^ (8)

·OH + ·OH → H_2_O_2_ (9)

Ca(OH)_2_ and Zn ions in water react with H_2_O_2_ generated through plasma to form hydroxide through the following reaction [43]. After heat treatment at 780 °C, it is converted to oxide.

Ca(OH)_2_ → Ca^2+^ + 2OH^−^ (10)

Ca^2+^ + 2Zn^2+^ + 3H_2_O_2_ → CaZn_2_(OH)_6_ (11)

CaZn_2_(OH)_6_ → CaO + 2ZnO + 3H_2_O (12)

Mg(OH)_2_ → MgO (13)

In this reaction equation, Ca and Zn react in an ionic state at a ratio of 1:2 and then are converted to CaO and ZnO. Residual Ca^2+^ ions that did not react during the decomposed Ca(OH)_2_ remain in the solution. Figure S1 and the reaction equation show that Mg does not participate in the reaction because the Ca content is high and the reactivity of Mg is lower than that of Ca.

**Figure 2:**
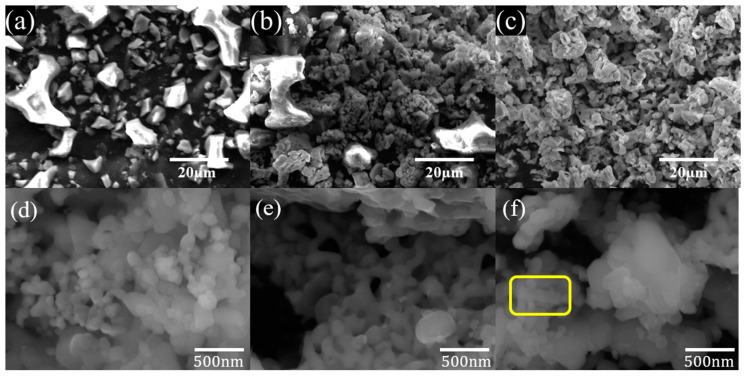
SEM image of (**a**) SF, (**b**) SF700, (**c**) SF900 (×3k), (**d**) SFZn1, (**e**) SFZn2, and (**f**) SFZn3 (×100k).

Scanning electron microscope images investigated the morphology of the as-prepared catalysts (Figure 2). Figure 2a shows a nonuniform, non-porous, sharp shape in SF. When SF was calcinated at 700 °C, the catalyst exhibited a sharp shape and porous particles, as shown in Figure 2b. The XRD data showed that SF (a) was present only with MgCaCO_3_, and for SF700 (b) it was only partially converted to MgO and CaO, and some MgCaCO_3_ remained unconverted. In Figure 2c, the catalyst shows uniform size and porous particles, which contrasts greatly with the samples SF and SF700. The uniform and porous structure provided a sufficient active surface area, indicating that sufficient conversion of MgCaCO_3_ to MgO and CaO occurred.

The plasma applied to the zinc-doped catalyst was significantly smaller than SF900. These particles grow to form aggregates with regular and smooth surfaces. As the Zn content increases, the overall particle size distribution tends to increase, and fine particles that did not grow sufficiently with small spherical aggregation are observed in SFZn1 (d). Still, spherical particles of uniform size are aggregated in SFZn2 (e). On the other hand, in the case of SFZn3 (f), the particles are aggregated to form uneven particles, and at the same time, due to the excessive content of Zn, a hexagonal crystal structure, which is the crystal structure of ZnO [44], appeared (yellow circle at Figure 2f). The distribution of constituent elements was determined through EDS mapping (Figures S2–S4). Overall, it was evenly distributed, but some locations of Mg and Zn were separated. From the reaction path Equations (3)–(13), MgO does not react significantly with Zn during the plasma process and is maintained as MgO. This mechanism can explain why the concentration of Zn and Mg is not uniform. From the EDS mapping of SFZn3 (Figure S4), ZnO is partially concentrated and separated from CaO compared to SFZn1 and SFZn2. This phenomenon can be described as a supersaturated state in which the solution added to this particular sample contains a large amount of Zn^2+^ during the preparation of the sample, so it does not react with Ca through plasma and precipitates itself to represent the form of ZnO [44].

Table 2 summarizes the characteristics related to the surface area of the prepared catalyst. On the surface area (S_BET_), the higher this value, the larger the active surface area of the catalyst, and it increased when Zn was doped, especially in SFZn2. The pore volume (V_p_) is the ability of the catalyst to adsorb a specific gas or liquid, and the larger the value, the greater the adsorption capacity. SFZn2 is a catalyst with the largest BET surface area and a high pore volume, suggesting that the active surface area and adsorption capacity are the highest. On the other hand, SFZn3 tended to be inferior to SFZn2 even though more Zn was loaded due to the precipitation of ZnO and densification of particles, as confirmed in the SEM image.

### 3.2. Structural and Chemical Properties

**Figure 3:**
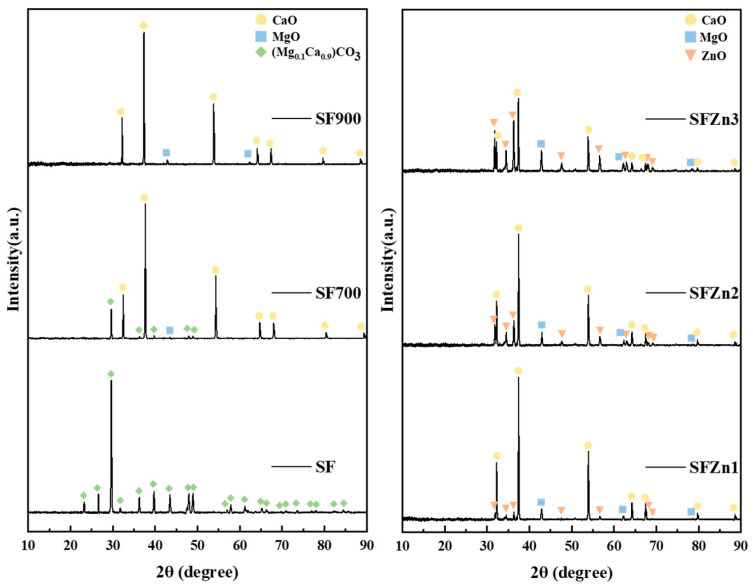
XRD patterns of the prepared catalysts.

X-ray diffraction (XRD) analysis was conducted to study the structure and crystallinity of the catalysts. In the XRD pattern of SF, Mg_0.1_Ca_0.9_CO_3_ peaks were detected. As the heat treatment temperature increased, the peaks became more apparent due to successfully converting CaO and MgO. As shown in Figure 3, starfish consists of the main crystalline phases of Mg_0.1_Ca_0.9_CO_3_ at 29.6°, 36.3°, 39.7°, 43.5°, 47.9°, and 48.9° at 2θ angles, confirming that the starfish used in this study was composed of Mg_0.1_Ca_0.9_CO_3_ (JCPDS # 071-1663). In SF700, which was subjected to heat treatment at 700 °C, cubic CaO (2θ = 32.2°, 37.3°, and 53.8°) and cubic MgO (2θ = 42.9° and 62.3°) were formed, and some MgCaCO_3_ remained unconverted. In the case of the XRD pattern of SF900, no Mg_0.1_Ca_0.9_CO_3_ peaks were detected, and diffraction patterns of CaO (JCPDS # 01-070-4068) and MgO (JCPDS # 01-089-4248) indicate that the conversion was optimized at 900 °C.

The XRD patterns of SFZn1 primarily showed peaks corresponding to CaO and MgO, with relatively low-intensity attributed to hexagonal ZnO (JCPDS # 01-080-0075) at 2θ values of 31.8°, 34.4°, and 36.3°. This suggests that ZnO is present in SFZn1 in a very small amount, as indicated by the low intensity of its peaks. In SFZn2 and SFZn3, the XRD patterns revealed more intense ZnO peaks than SFZn1, indicating a higher loading of ZnO in these samples. In particular, as the amount of Zn increased, the peak intensity of CaO decreased, suggesting that Ca and Zn react 1:2 during the reaction, and calcium remains in the water in an ionic state after the reaction completes, where both CaO and ZnO are precipitated. In addition, as shown in the SEM image, due to the precipitation of ZnO, the ZnO peak was prominent in SFZn3, and the CaO peak was comparatively weaker than other samples.

**Figure 4:**
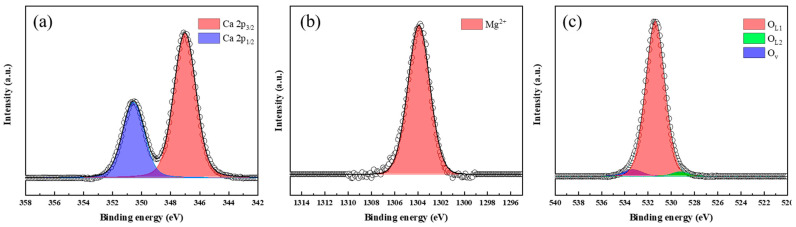
XPS spectra of SF900: (**a**) Ca 2p, (**b**) Mg 1s, and (**c**) O 1s.

The surface chemical states of the samples were characterized based on the XPS analysis. Figure 3 shows the core level of Ca 2p, Mg 1s, and O 1s spectra of SF900. The binding energy of Ca 2p (Figure 4a) has two asymmetric peaks: Ca 2p_3/2_ around 347 eV and Ca 2p_1/2_ around 350.4 eV. The Ca peak appeared at a ratio of 2:1, with a difference of about 3.4 eV. [45]. In Figure 4b, Mg peaks were ascribed to its oxide state (Mg^2+^, at 1304 eV) [46]. The core level of O 1s peak of SF900 can be deconvoluted in three components at 530.4 eV, 531.4 eV, and 533.4 eV. O _L1_ (531.4 eV), and O _L2_ (530.4 eV) is the oxygen in the lattice, constituting oxides of CaO [47] and MgO [48], respectively. O_v_ indicates the oxygen peak caused by water adsorbed on the surface of the catalyst [49,50]. The overall surface area and basic sites increased by adding Zn through the plasma process, so the water adsorption increased.

**Figure 5:**
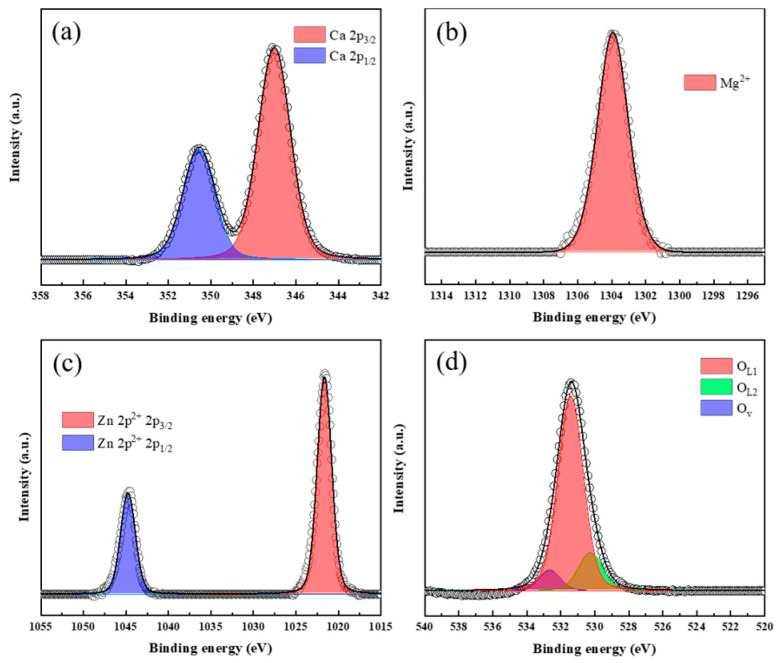
XPS spectra of SFZn2: (**a**) Ca 2p, (**b**) Mg 1s, (**c**) Zn 2p, and (**d**) O 1s.

Figure 5 shows the detailed analyses of the XPS narrow scan of SFZn2. In the figure, Ca 2p showed two distinct peaks of 2p_3/2_ at 347 eV and 2p_1/2_ at 351.4 eV, similar to SF900 without Zn addition. Also, Mg 1s was found to be 1304 eV for Mg ^2+^. On the other hand, the Zn 2p peak appeared as dual peaks between 1015 and 1055 eV (Figure S5). The difference due to the formation of ZnO can be seen in O 1s, where the intensity of O_L2_ increased due to the oxide peak of ZnO corresponding to 530.4 eV including MgO, and O_L1_, the oxide lattice peak of CaO, shifted to 531.8 under the influence of ZnO [28]. The XPS spectra for SFZn1 and SFZn3 are presented in the Supplementary Materials (Figures S6 and S7).

Table 3 shows the element ratio of the prepared catalysts calculated by ICP analysis. In general, the ratio of Ca and Mg varies depending on the size of the starfish from 10:1 to 20:1, as reported by Brügmann L et al. [20]. The amount of Zn added with SFZn1: SFZn2: SFZn3 is 1:2:4 under the experimental conditions, and in the ICP results, Zn increases at the same ratio as the amount added. Based on the previous discussion and reaction (3–13), Ca and Zn participated in the plasma process at a ratio of 1:2, and unreacted Ca ions remained in the solution with the introduction of Zn. Therefore, as shown in the ICP results, as the content of Zn increases, the content of Ca decreases. As a result, the total mass of the final product is reduced, while the amount of Mg is relatively large because Mg does not participate in the reaction and remains intact. This trend is particularly significant in SFZn3, where precipitation of ZnO appears.

The basic strength of the catalytic active site is an important indicator in biodiesel production. As previously reported, an increase in the amount of Zn affects the basic strength of the catalyst [29]. The Lewis base site was formed under Zn added to the existing CaO surface. The basicity of the as-prepared catalysts was calculated using Equation (14) [51,52,53].

Basicity (mmol/g) = (Volume of HCl (L) × Molarity of HCl (mol/L))/(Mass of catalyst (g))(14)

The calculated basicity based on HCl titration is 2.48, 2.76, 3.04, and 2.77 mmol/g for SF900, SFZn1, SFZn2, and SFZn3 samples, correspondingly. As shown in Table 4, the basicity increased in the order of SFZn2, SFZn3, SFZn1, and SF900. Among all catalysts, SFZn2 has the highest surface area and pore volume and has demonstrated the highest basic strength and site density. On the other hand, SFZn3 shows a lower basic strength with increasing Zn content due to precipitation, as depicted in Figure 2f and the previous discussion. The site density of each catalyst was then calculated based on its mass and basic strength. SFZn3 showed similar basic strength despite having a much wider surface area than SFZn1. This phenomenon is caused by ZnO precipitation, and since ZnO has a lower alkalinity than CaO, the basic strength of SFZn3 becomes low.

### 3.3. Catalytic Performance

**Figure 6:**
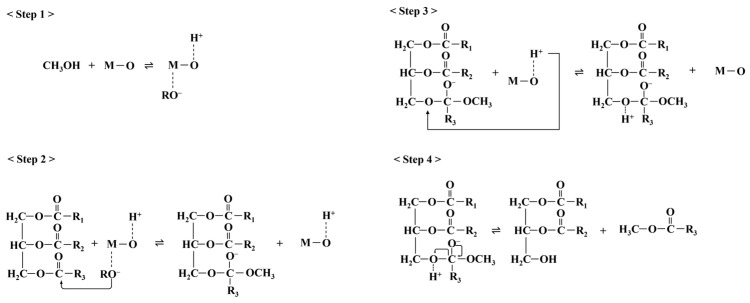
Possible mechanisms of the transesterification reaction with catalysts.

Figure 6 shows the possible mechanism of the catalyst in the transesterification reaction. The M is Lewis’s basic site of catalyst. In the first step, the catalyst reacts with methanol, forming the methoxide anion. Then, the methoxide anion attacks the carbonyl carbon of triglycerides to form an alkoxycarbonyl intermediate (step 2). This intermediate reacts with H^+^ derived from CaO-H^+^ to generate another alkoxycarbonyl intermediate and CaO (step 3). Finally, the intermediate divides into biodiesel (methyl ester) and diglycerides (step 4) [54,55].

**Figure 7:**
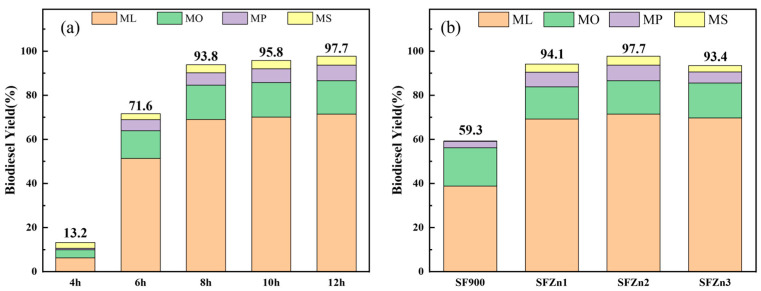
Biodiesel yield of grape seed oil via transesterification under 68 °C: (**a**) comparison with different reaction times of SFZn2, and (**b**) transesterification of each sample for 12 h.

Figure 7 shows the biodiesel yield of grape seed oil with different transesterification times of SFZn2 (a) and transesterification of each sample for 12 h under 68 °C (b), where methyl linoleate, methyl oleate, methyl palmitate, and methyl stearate are indicated as ML, MO, MP, and MS, respectively. All reactors were placed in a water bath for the transesterification reaction, and the reaction time was measured after the water bath temperature reached 68 °C. The position of the peak according to the retention time analyzed through HPLC can be confirmed in the Supplementary Materials (Figures S8–S10). When transesterification was performed for 4 h, the total yield was very low at 13.2%, but from 6 h, a yield of 71.6% was obtained. After that, more than 90% of conversions proceeded from 8 h, 95.8% at 10 h, and 97.7% at the maximum value of 12 h. Major products of grape seed oil are linoleic acid, oleic acid, palmitic acid, and stearic acid, which are 74.2%, 14.8%, 7.2%, and 3.8% of the composition, respectively. The composition of grape seed oil is summarized in Table 1. As all the acids convert into methyl esters, the composition of the esters turns into 74.6% for ML, 15.0% for MO, and 6.7% and 3.8% for MP and MS.

Each detailed methyl ester tended to increase as the reaction time increased, but methyl oleate did not increase significantly from 8 h. When comparing the yield of each methyl ester at 12 h with the ratio of representative fatty acids constituting grape seed oil shown in Table 1, it was almost similar, indicating that most of the triglycerides were converted to esters when transesterification was performed for 12 h through a catalyst. The detailed conversion of each product over time is summarized in Table S1.

Figure 7b shows the results of the grape seed oil transesterification reaction using each catalyst. Methyl linoleate was converted the most, and methyl stearate was converted the least, showing a similar trend as shown in Table 1. In the case of SF900, the overall yield was the lowest, and the stearic acid was hardly converted to methyl stearate. As the amount of Zn increased, the yield of each component increased, and the yield of SFZn1 was 94.1%, SFZn2 was 97.7%, and SFZn3 was 93.4%. The catalyst with the highest Zn loaded was SFZn3, but the result of transesterification showed the highest efficiency in SFZn2. Due to the influence of Zn on the CaO surface with the highest surface area value, SFZn2 showed the best performance in the transesterification reaction. In the case of SFZn3, despite exhibiting higher basic strength and specific area than SFZn1, the yield of SFZn3 is comparatively lower than that of SFZn1 because of the ZnO formation. The biodiesel conversion yield showed the same trend, as summarized in Table 5.

**Figure 8:**
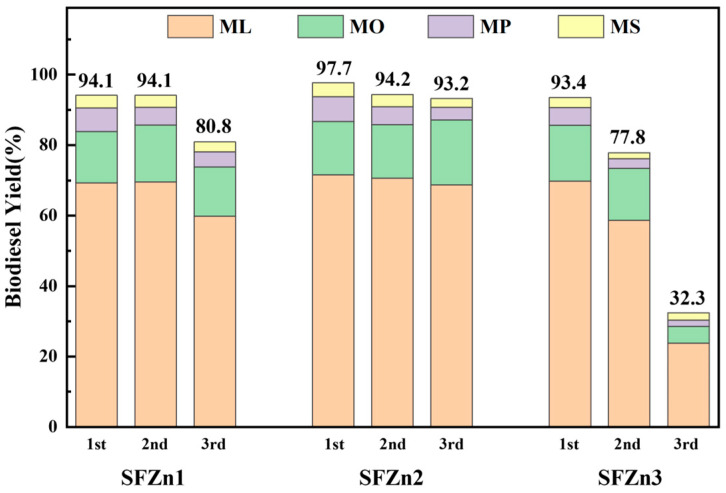
Biodiesel yield of grape seed oil of each catalyst in three consecutive runs (12 h, 68 °C).

The recyclability of each catalyst was evaluated by three consecutive runs. Figure 8 shows the components of each ester as abbreviations ML, MO, MP, and MS. In the case of SFZn1, methyl linoleate and methyl oleate slightly increased in the first and second cycles, but the overall yield was almost similar at 94.1%. Still, in the third cycle, the yield of methyl linoleate and methyl oleate also decreased, and the overall yield decreased to 80.8%. In the case of SFZn2, the yield gradually decreased according to the number of recycles, but it recorded a yield of 93.2% even after three uses, showing the best durability. On the other hand, SFZn3 decreased with a yield of 29% from the second time and further reduced to 32.3% in the third run. As discussed in the previous SEM image, ZnO is presented as a column-like structure in SFZn3. This structure formed due to the precipitation of ZnO. As a result, SFZn3 results in poor stability and possible Zn or Ca metals leaching during the transesterification reaction. The detailed biodiesel products of all catalysts in each run are summarized in Table 6. To prove the advantage of plasma-modified catalysts, the performance of the catalyst produced through the hydrothermal synthesis method (convention method) was prepared at the ratio of SF: Zn = 10:1 and compared to SFZn2 with identical transesterification conditions. Details of hydrothermal synthesis and its performance are summarized in Table S2 in the Supplementary Materials. While the maximum yield was 97.7% for the plasma-synthesized catalyst (SFZn2), which was maintained at over 90% after the subsequent reuse tests, the catalyst fabricated via hydrothermal synthesis showed a significantly lower performance of 70.9%, and the yield decreased rapidly to 23.4% for the second use. In the conventional hydrothermal process, Zn addition is based on the thermal chemical reactions. On the other hand, plasma modification applies highly active electrons and radicals generated through plasma-induced reactions that are further added to the substrate, as shown in Equations (3)–(13). Based on the result, it can be confirmed that Zn addition via plasma-induced reactions is much more effective and can maintain higher stability compared to catalysts fabricated from the hydrothermal method.

## 4. Conclusions

Biomass-based catalysts were developed by applying dried starfish, a type of marine waste, as a catalyst substrate material for the conversion of grape seed oil to biodiesel. We improved the basicity and number of active sites of the catalysts by increasing the surface area by Zn addition via plasma engineering. Through XRD and XPS surface analyses, we confirmed that CaO, MgO, and ZnO were formed on all catalyst surfaces. As the amount of Zn increases, surface area and basic strength tend to enhance. However, precipitation of ZnO occurs when the substrate to ZnO ratio increases to 5:1 (SFZn3), resulting in adverse effects where surface area and basic strength decrease sharply and result in low catalytic performance and stability of the catalyst. The plasma-modified catalyst was optimized with a Zn to Mg_0.1_Ca_0.9_CO_3_ ratio of 10:1 (SFZn2). The conversion of grape seed oil to methyl ester was as high as 97.7% under 12 h transesterification. In addition, the performance of the SFZn2 catalyst maintained over 93.2% even after three repeated runs. On the other hand, in the case of SFZn3, where ZnO precipitation occurred, the stability was significantly reduced, and the yield decreased to 32.3% after three reused tests. This paper provides significant insight into the novel synthesis method of marine waste catalysts for low-cost and sustainable biodiesel production.

## Figures and Tables

**Table 1:** Representative fatty acid content per 1 g of grape seed oil.

Linoleic Acid	Oleic Acid	Palmitic Acid	Stearic Acid
694.55 mg (74.2%)	139.38 mg (14.8%)	61.71 mg (7.2%)	35.69 mg (3.8%)

**Table 2:** Textural properties of catalysts.

Catalyst	S_BET_ (m^2^/g)	V_p_ (cm^3^/g)	Pore Size (Å)
SF900	12 ± 5%	0.031 ± 5%	106 ± 5%
SFZn1	16 ± 5%	0.032 ± 5%	81 ± 5%
SFZn2	29 ± 5%	0.052 ± 5%	73 ± 5%
SFZn3	23 ± 5%	0.045 ± 5%	77 ± 5%

**Table 3:** ICP data of the prepared samples.

Catalyst	at. %			wt. % (RSD, %)		
	Ca	Mg	Zn	Ca	Mg	Zn
SF900	91.24	8.76	0	94.50 (0.53)	5.50 (0.47)	0
SFZn1	79.81	13.81	6.38	80.95 (0.64)	8.49 (0.51)	10.56 (0.74)
SFZn2	70.75	14.51	14.74	68.30 (0.81)	8.49 (0.85)	23.21 (0.82)
SFZn3	51.12	22.17	26.71	47.27 (0.36)	12.44 (0.44)	40.29 (0.35)

**Table 4:** Calculated basic strength and site density of prepared catalysts.

	SF900	SFZn1	SFZn2	SFZn3
Basic strength (mmol/g)	2.48	2.76	3.04	2.77
Site density (sites/g)	1.49 × 10^21^	1.66 × 10^21^	1.83 × 10^21^	1.67 × 10^21^

**Table 5:** Biodiesel yields through transesterification of the prepared catalysts (12 h, 68 °C).

Catalyst	Yield (%)				Total Yields (%)
	Methyl Linoleate	Methyl Oleate	Methyl Palmitate	Methyl Stearate	
SF900	38.8	17.4	2.9	0.2	59.3
SFZn1	69.2	14.6	6.7	3.6	94.1
SFZn2	71.4	15.3	7	4	97.7
SFZn3	69.7	15.8	5	2.9	93.4

**Table 6:** Summary of the production yield of each catalyst in three consecutive runs for 12 h, 68 °C.

Catalyst		Yield (%)				Total Yields (%)
		Methyl Linoleate	Methyl Oleate	Methyl Palmitate	Methyl Stearate	
SFZn1	1st run	69.2	14.6	6.7	3.6	94.1
	2nd run	69.4	16.3	5	3.4	94.1
	3rd run	59.7	14	4.3	2.8	80.8
SFZn2	1st run	71.4	15.3	7	4	97.7
	2nd run	70.5	15.2	5.1	3.4	94.2
	3rd run	68.6	18.5	3.6	2.5	93.2
SFZn3	1st run	69.7	15.8	5	2.9	93.4
	2nd run	58.5	14.8	2.8	1.7	77.8
	3rd run	23.6	4.8	1.7	2.2	32.3

## Data Availability

The original contributions presented in the study are included in the article/Supplementary Material, further inquiries can be directed to the corresponding authors.

## Supplementary Materials

The following supporting information can be downloaded at https://www.mdpi.com/article/10.3390/nano14151313/s1, Figure S1: The XRD data of SFZn2 before heat treatment; Figure S2: The EDS image of SFZn1; Figure S3: The EDS image of SFZn2; Figure S4: The EDS image of SFZn3; Figure S5: XPS spectra of the prepared sample; Figure S6: The XPS spectra of the SFZn1; Figure S7: The XPS spectra of the SFZn3; Figure S8: HPLC data for different transesterification time under 68 °C of SFZn2; Figure S9: HPLC data for 12 h transesterification under 68 °C. (SF900: Non-dilution, SFZnx: Dilution ration 1:5 to mobile); Figure S10: HPLC data for 3 times recycle of catalysts (12 h, 68 °C); Figure S11: HPLC data for 2 times recycling of a catalyst via hydrothermal treatment (68 °C for 12 h); Table S1: Biodiesel yield through transesterification with different transesterification time under 68 °C; Table S2: Biodiesel yield via transesterification for 12 h under 68 °C with hydrothermal treated catalyst.
