# Supplementary material for: Plasma Modification of Biomass-Based Starfish Catalysts for Efficient Biodiesel Synthesis
Source: Nanomaterials (Basel). 2024 Aug 4;14(15):1313. doi: 10.3390/nano14151313 (PMC11313850; doi:10.3390/nano14151313)
Supplement: Supplementary file 1 [file nanomaterials-14-01313-s001.zip › nanomaterials-3107740-supplementary.pdf]

Supplementary Materials

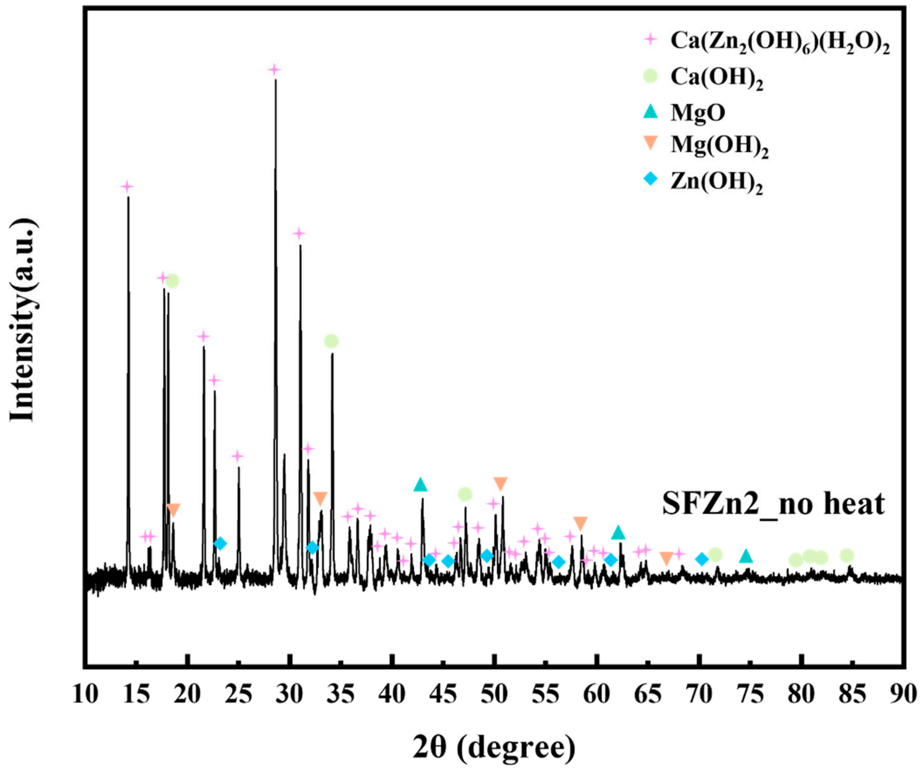

Figure S1. The XRD data of SFZn2 before heat treatment.

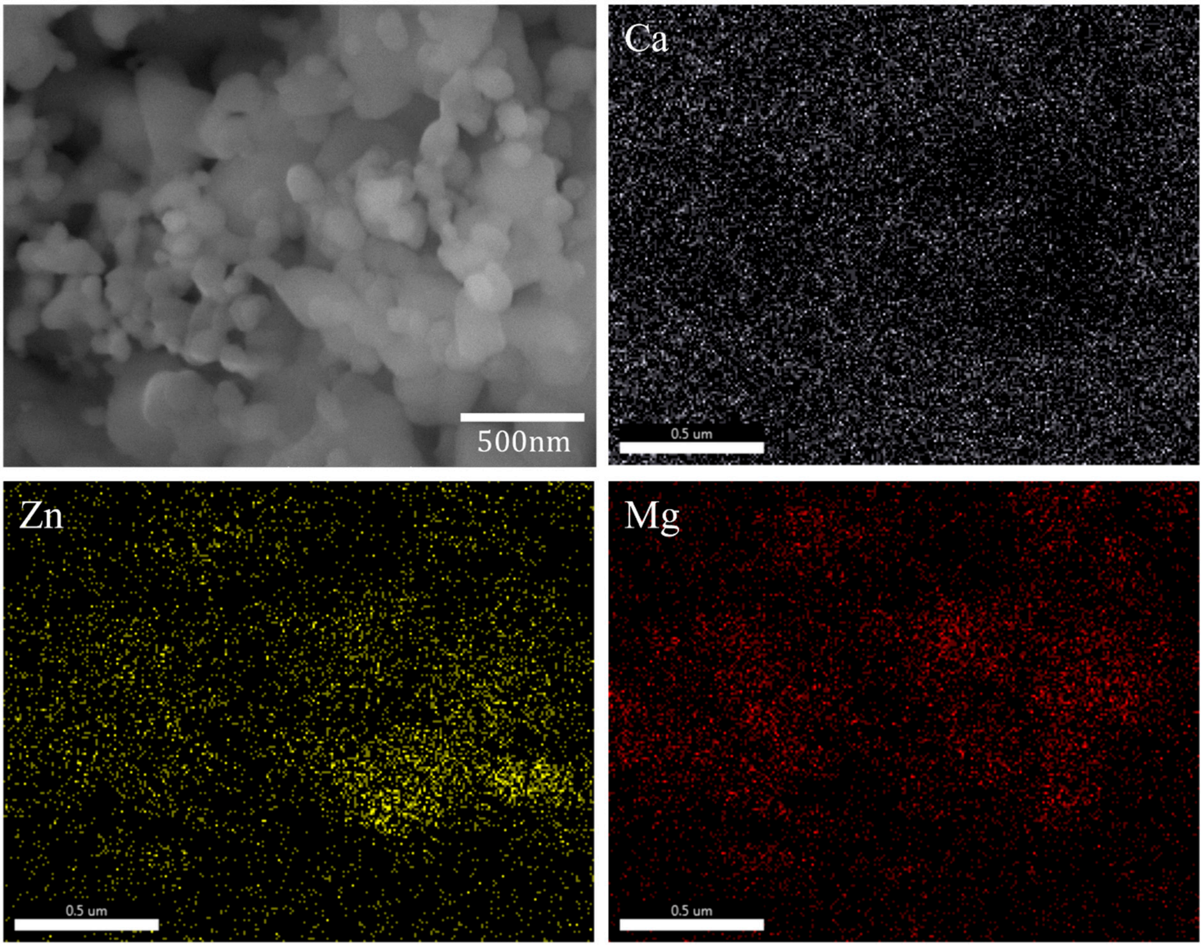

Figure S2. The EDS image of SFZn1.

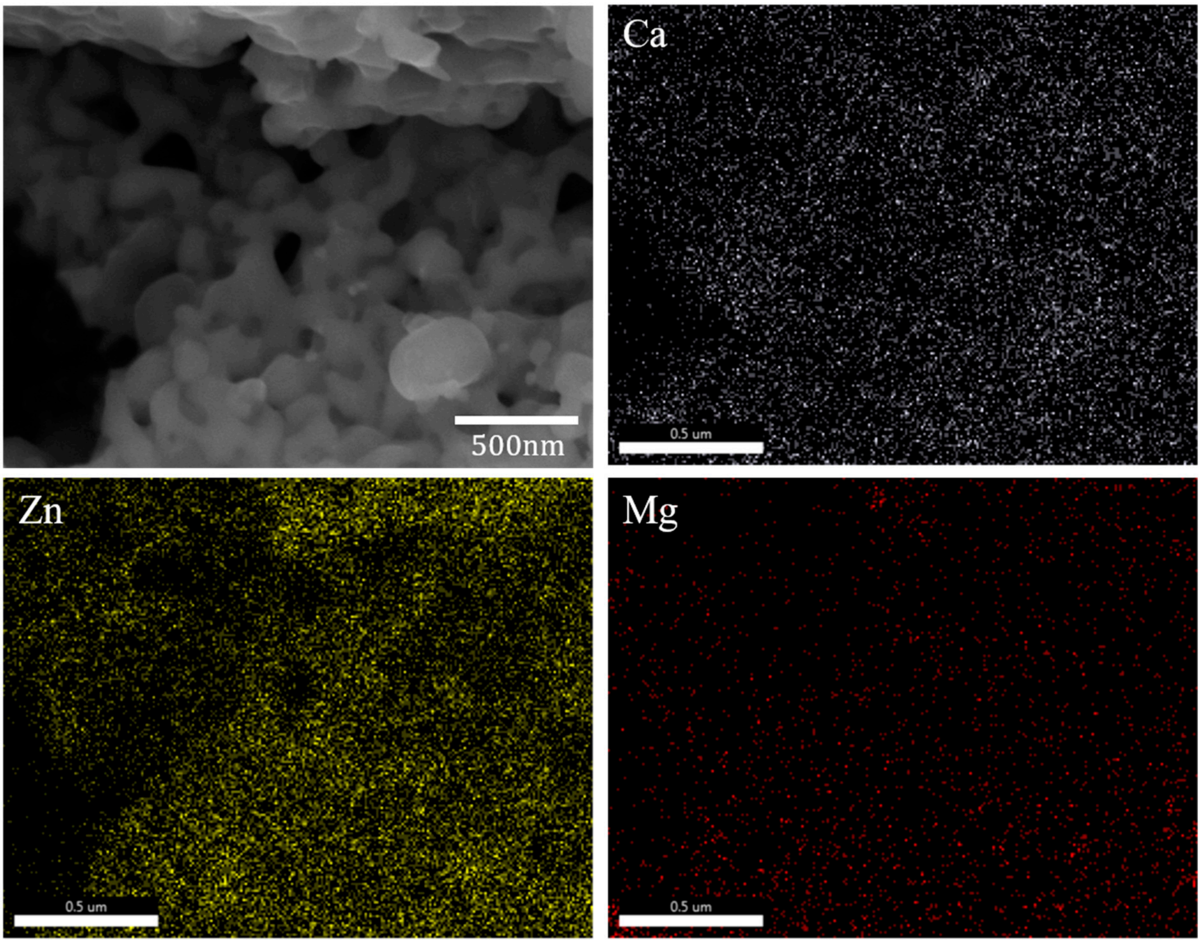

Figure S3. The EDS image of SFZn2

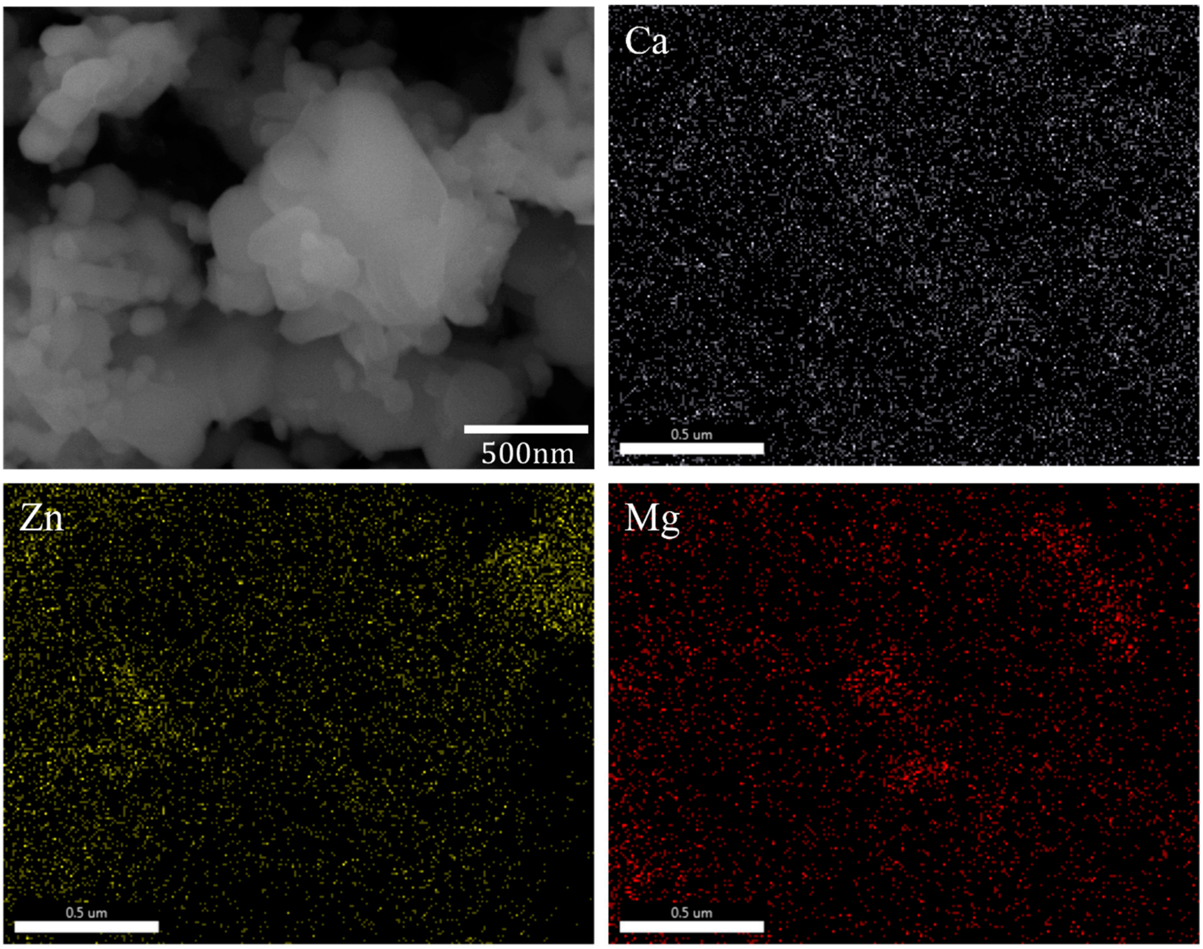

Figure S4. The EDS image of SFZn3

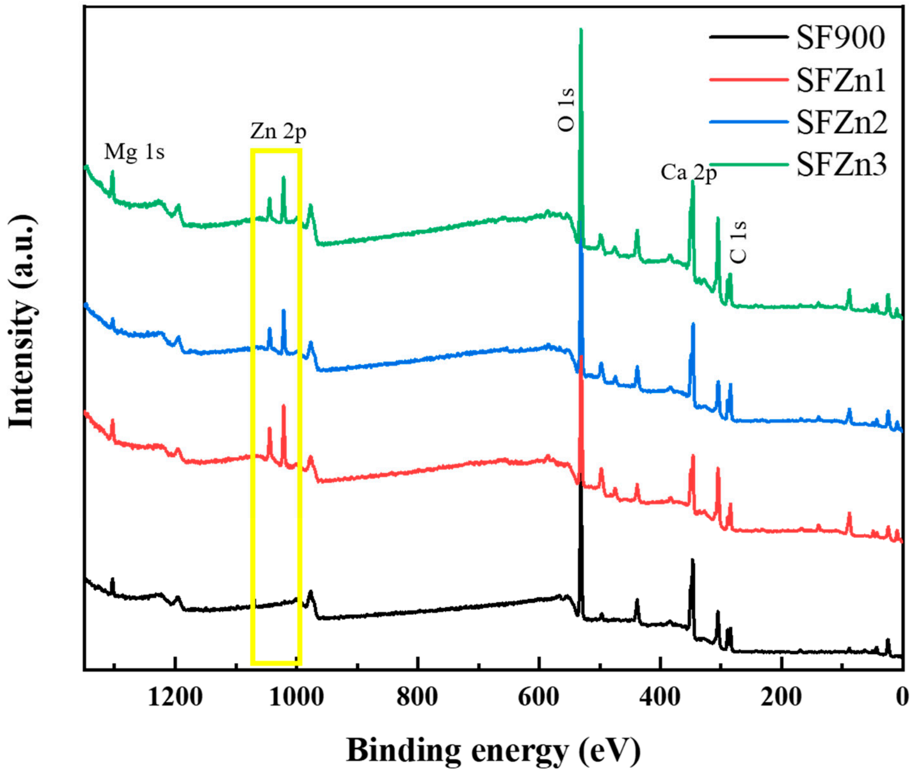

Figure S5. XPS spectra of the prepared sample

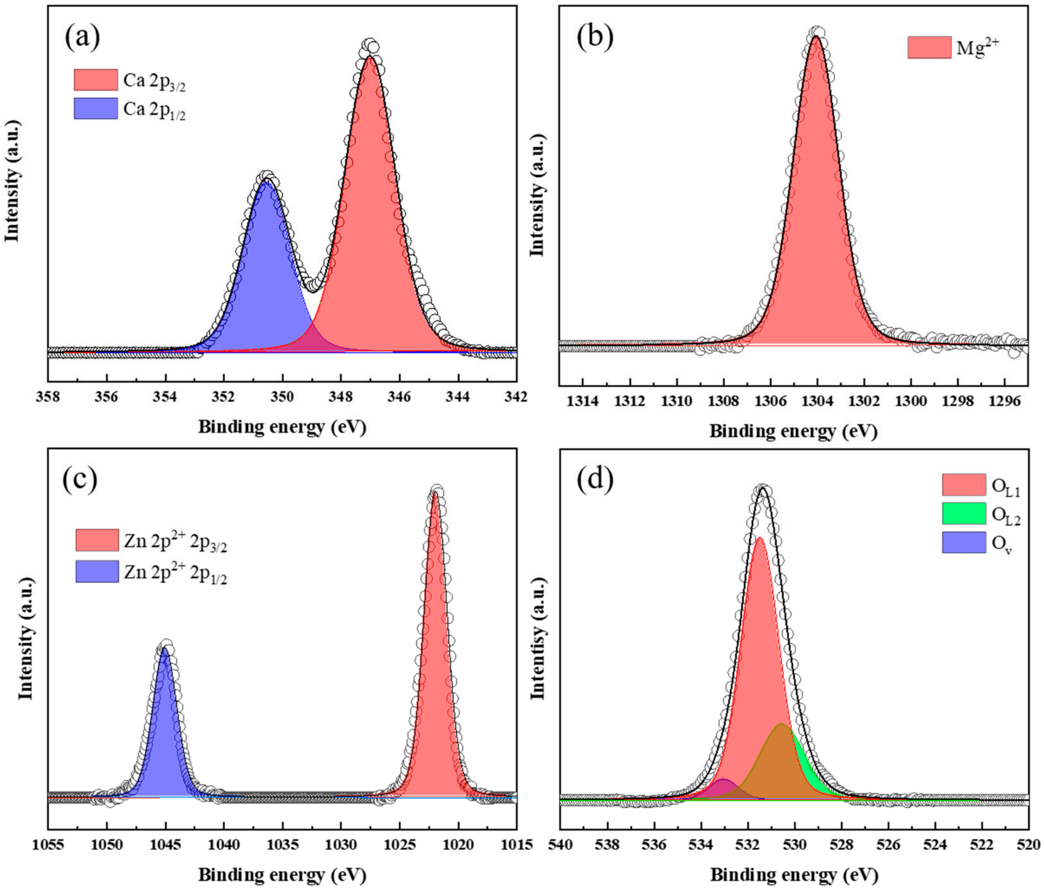

Figure S6. The XPS spectra of the SFZn1 (a) Ca 2p, (b) Mg 1s, (c) Zn 2p, (d) O 1s

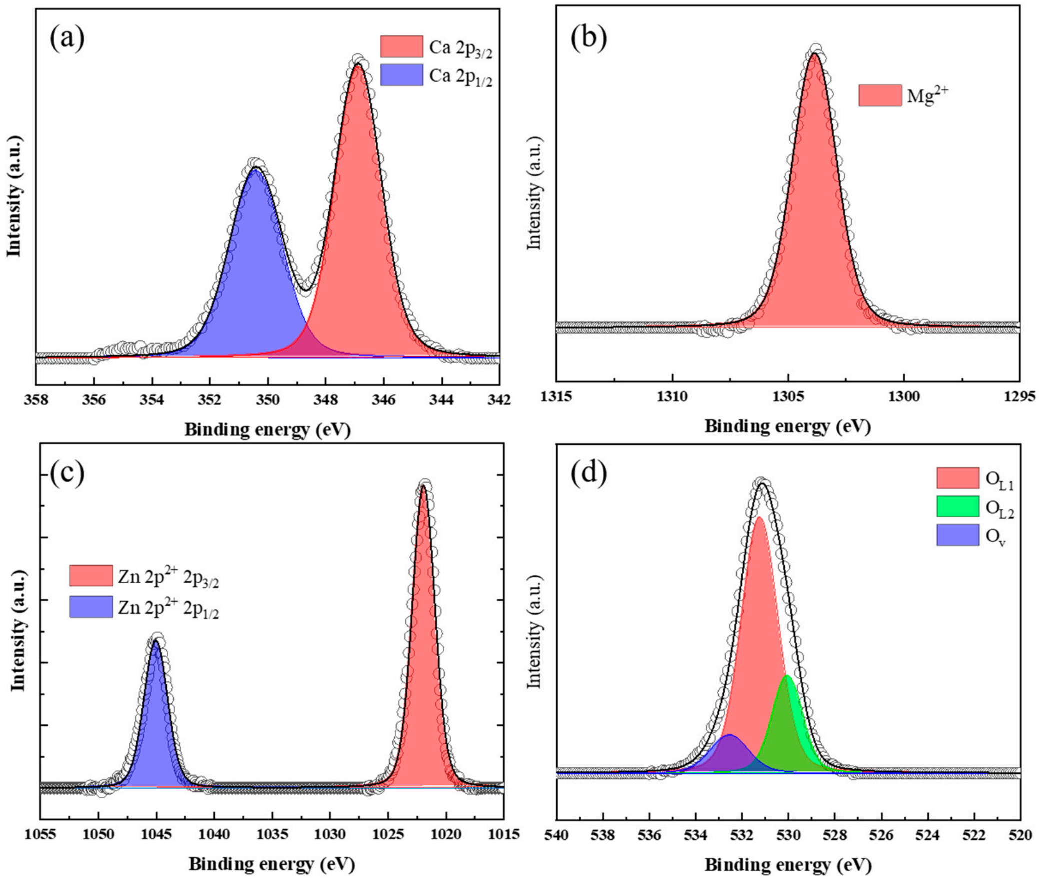

Figure S7. The XPS spectra of the SFZn3 (a) Ca 2p, (b) Mg 1s, (c) Zn 2p, (d) O 1s

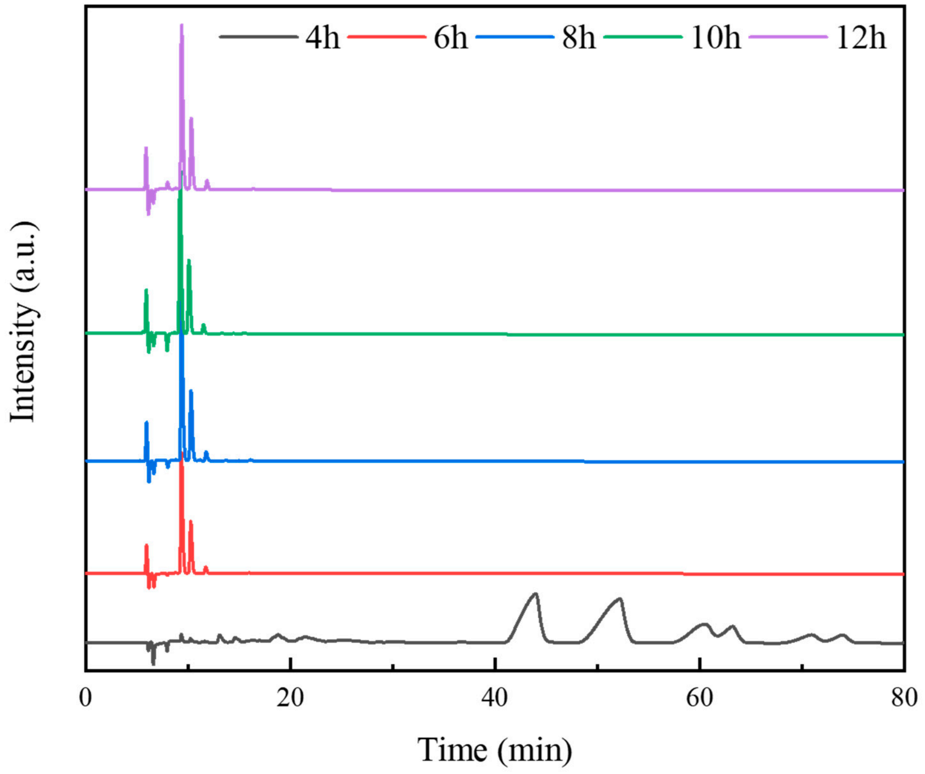

Figure S8. HPLC data for different transesterification time under 68 ℃ of SFZn2.

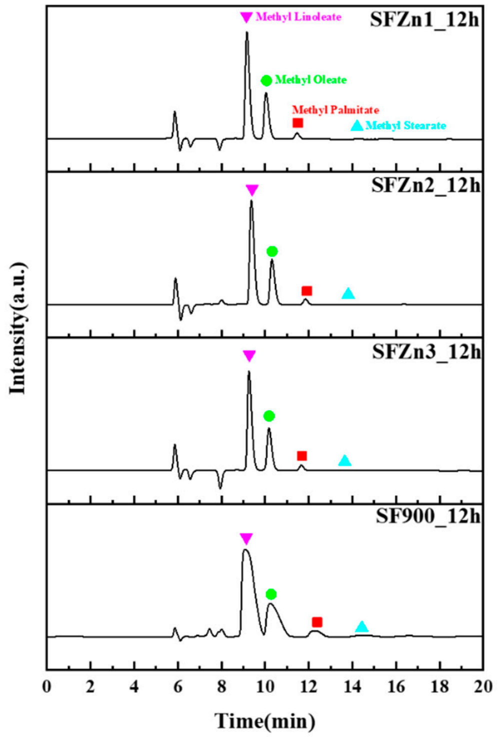

Figure S9. HPLC data for 12h transesterification under 68 ℃. (SF900: Non-dilution, SFZnx: Dilution ratio 1:5 to mobile

phase)

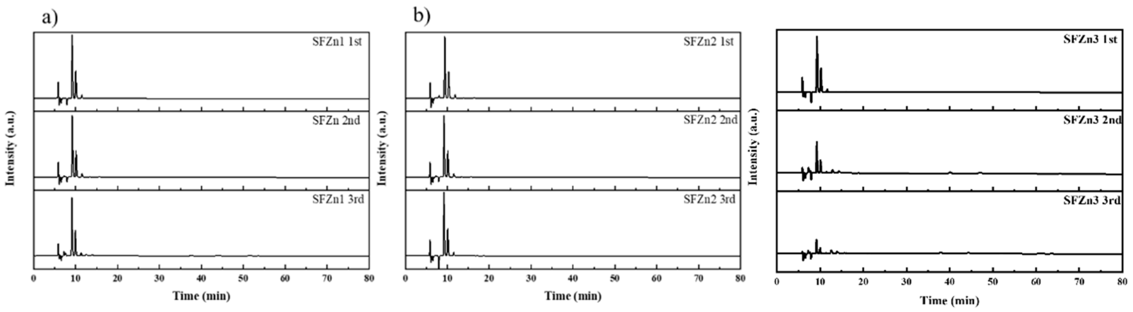

Figure S10. HPLC data for 3 times recycle of catalysts (12 h, 68 ℃) (a) SFZn1, (b) SFZn2, (c) SFZn3.

Table S1. Biodiesel yield through transesterification with different transesterification time under 68 ℃.

**Catalyst**	**Yield**				**Total Yields (%)**
	**Methyl Linoleate (%)**	**Methyl Oleate (%)**	**Methyl Palmitate (%)**	**Methyl Stearate (%)**	
4 h	6.3	3.6	0.7	2.6	13.2
6 h	51.4	12.5	5	2.7	71.6
8 h	69	15.5	5.6	3.7	93.8
10 h	70.1	15.6	6.3	3.8	95.8
12 h	71.4	15.3	7	4	97.7

Synthesis Hydrothermal process catalysts

A molar ratio of 10:1 for SF900 and Zinc acetate dihydrate was placed in each beaker and stirred at 600 rpm with 100 deionized water for 2 h. After stirring, both solutions were added to a Teflon-lined autoclave with 0.1 M ammonia solution. Then, Hydrothermal treatment proceeded at 120 °C for 12 h, and filtration was followed. For the last step, drying at 105 °C oven for 3 h and calcined at 850 °C for 2 h [36,37].

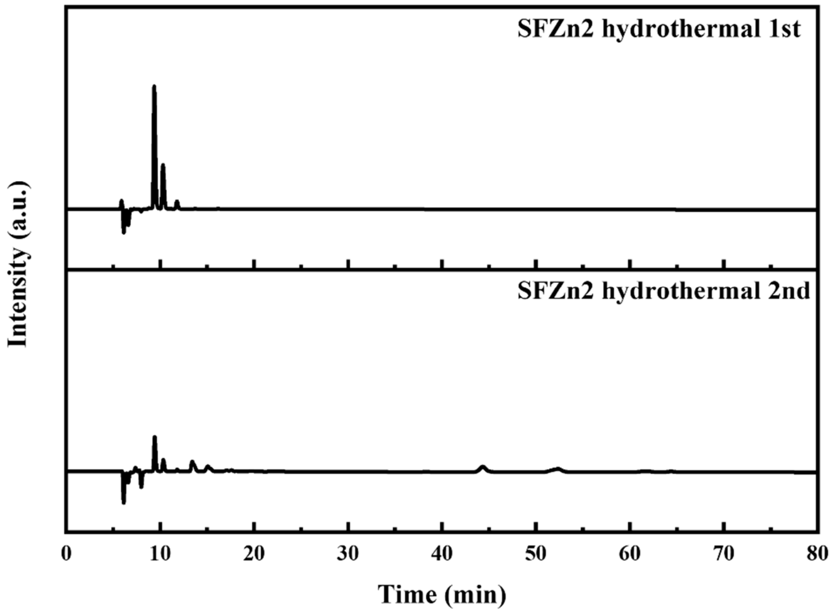

Figure S11. HPLC data for 2 times recycling of a catalyst via hydrothermal treatment (68 ℃ for 12 h)

Table S2. Biodiesel yield via transesterification for 12 h under 68 °C with hydrothermal treated catalyst

**Catalyst**	**Yield**				**Total Yields (%)**
	**Methyl Linoleate (%)**	**Methyl Oleate (%)**	**Methyl Palmitate (%)**	**Methyl Stearate (%)**	
Hydrothermal 1st	47.3	13.1	3.96	1.9	72.3
Hydrothermal 2nd	15.4	12.8	3.42	1.8	33.4
